# Analytical cross-sectional study on lifestyle factors associated with chronic migraine frequency

**DOI:** 10.6026/973206300212922

**Published:** 2025-08-31

**Authors:** Sahil Rameshbhai Kalsariya, Abhishek Palaniappan, Ajeet Saoji, Sailesh I.S Kumar, Jagriti Relhan

**Affiliations:** 1 Department of Medicine, Shalby Hospital, Ahmedabad, Gujarat, India; 2 Department of General Medicine, KMCH Institute of Health Science and Research, Coimbatore, Tamil Nadu India; 3 Department of Community Medicine, NKP Salve Institution of Medical Sciences and Research Centre, Nagpur, Maharashtra, India; 4 Department of General Medicine, Madras Medical College, Tamilnadu, India; 5 Department of Medicine, Kasturba Medical College Manipal, Karnataka, India

**Keywords:** Chronic migraines, lifestyle factors, cross-sectional study, sleep quality, stress, caffeine, physical activity

## Abstract

The relationship between lifestyle factors and migraine frequency in 130 adults diagnosed with chronic migraine is of interest. Participants completed validated questionnaires assessing diet, physical activity, sleep and caffeine intake and stress levels. Poor sleep quality, high stress and excessive caffeine intake were significantly associated with increased migraine frequency. Regular physical activity and consistent meal patterns were inversely related to migraine days per month. Identifying modifiable lifestyle factors may help guide non-pharmacological management of chronic migraine.

## Background:

Migraine, characterized by recurrent and debilitating headaches often accompanied by sensory disturbances known as "aura", represents a significant global health challenge [1]. These headaches can manifest unilaterally or bilaterally and are frequently associated with visual disturbances, such as flashing lights and auditory symptoms [2]. The timing of the aura can vary, occurring either before the onset of a headache or even after it has begun. Chronic migraine, defined as headaches occurring on 15 or more days per month for more than three months with migraine features on at least 8 days, poses a significant burden on individuals' quality of life, productivity and mental health [3]. While the etiology of chronic migraine is multifactorial, lifestyle factors such as sleep habits, stress, dietary triggers, physical inactivity and caffeine consumption are frequently implicated in exacerbating migraine frequency and severity [4]. Unlike episodic migraine, chronic migraine often reflects a sensitized central nervous system and identifying modifiable external contributors is crucial for both prevention and long-term management [5]. Although pharmacological interventions are central to treatment, growing attention has been placed on non-pharmacologic strategies, particularly lifestyle modifications, which are often underutilized in clinical practice [6]. Understanding the association between daily behaviors and migraine patterns can help identify high-risk profiles and guide individualized care plans [7]. Therefore, it is of interest to explore how specific lifestyle factors are associated with the frequency of migraine attacks in adults diagnosed with chronic migraine.

## Materials and Methods:

This analytical cross-sectional study included 130 adult patients aged between 18 and 55 years who were clinically diagnosed with chronic migraine according to the International Classification of Headache Disorders, 3rd edition (ICHD-3) criteria. Participants were recruited consecutively from neurology outpatient clinics and specialized headache centers over a period of four months. Individuals with other headache types, comorbid neurological disorders, major psychiatric illness, pregnancy, or recent head trauma were excluded to minimize confounding. Data collection was performed using a structured interview and self-administered questionnaires. Participants provided information regarding their headache frequency, severity and duration over the past three months, including the average number of migraine days per month. Lifestyle factors were assessed using validated tools: sleep quality and duration were evaluated using the Pittsburgh Sleep Quality Index (PSQI), physical activity was measured via the International Physical Activity Questionnaire (IPAQ) and psychological stress was assessed using the Perceived Stress Scale (PSS). Dietary habits, including meal regularity and known dietary triggers (e.g., processed foods, cheese, chocolate), along with daily caffeine intake (cups/day) and screen time (hours/day), were also recorded. Migraine frequency was categorized into low (15-19 days/month), moderate (20-24 days/month) and high (≥25 days/month). Descriptive and comparative statistical analyses were performed using SPSS software. Associations between lifestyle factors and migraine frequency were evaluated using chi-square tests and ANOVA, while multivariable logistic regression models were applied to adjust for age, gender, BMI and prophylactic medication use. Significance was set at a p-value of less than 0.05.

## Results:

Higher migraine frequency was significantly associated with poor sleep quality, high stress levels, irregular meals, excessive caffeine consumption and physical inactivity. Participants with healthy sleep patterns, regular physical activity and consistent dietary habits reported fewer monthly migraine days, suggesting a strong association between modifiable lifestyle behaviors and chronic migraine burden. Table 1 (see PDF) shows most participants were female, with an average age of 36 years; comorbid obesity and high stress levels were more common in the high-frequency group. Table 2 (see PDF) shows poor sleep quality was more prevalent in the high-frequency migraine group. Table 3 (see PDF) shows participants with higher migraine frequency had shorter average sleep duration. Table 4 (see PDF) shows stress levels, as measured by the perceived Stress Scale, were significantly higher in those with more frequent migraines. Table 5 (see PDF) shows higher caffeine intake was significantly associated with increased migraine frequency. Table 6 (see PDF) shows irregular meal patterns were common among those with more frequent migraines. Table 7 (see PDF) shows physical activity levels were inversely related to migraine frequency. Table 8 (see PDF) shows participants with high screen time reported more migraine days. Table 9 (see PDF) shows migraine frequency was significantly associated with multiple lifestyle factors in multivariable regression. Participants with healthier lifestyle profiles reported fewer migraine-related work absences. Table 10 (see PDF) shows that migraine frequency had a strong impact on occupational and daily activity impairment. Individuals in the low-frequency group (15-19 days/month) reported the fewest missed workdays (0.9 days/month) and the lowest rate of severe impairment (11.9%). The moderate-frequency group (20-24 days/month) missed significantly more days (2.4 days/month) with about one-third (32.6%) experiencing severe impairment. The high-frequency group (≥25 days/month) reported the greatest functional disability, with an average of 4.3 missed workdays/month and over half (54.8%) reporting severe impairment. The association was statistically significant (p < 0.001), highlighting the escalating work and activity burden with increasing migraine frequency.

## Discussion:

This analytical cross-sectional study highlights the significant associations between various modifiable lifestyle factors and chronic migraine frequency. Individuals experiencing higher migraine days per month were more likely to report poor sleep quality, elevated psychological stress, irregular meals, excessive caffeine intake, low physical activity levels and prolonged screen time [8]. These findings reinforce the multifactorial nature of chronic migraine and the importance of lifestyle habits in influencing its frequency and severity [9]. Among all lifestyle variables, poor sleep and high stress emerged as the most consistent and powerful predictors of high-frequency migraines [10]. These factors may contribute to cortical hyperexcitability and hypothalamic-pituitary-adrenal (HPA) axis dysregulation, both of which are implicated in migraine pathophysiology [11]. Sleep deprivation may also interfere with pain modulation mechanisms, increasing susceptibility to migraine triggers. Similarly, chronic stress can induce vasodilation and neuroinflammatory changes that exacerbate headache patterns [12]. The positive association between caffeine overuse and migraine frequency underscores the dual role of caffeine-as both a trigger and a withdrawal inducer-when consumed excessively or inconsistently [13]. Meanwhile, irregular meals and frequent skipping of breakfast may lead to fluctuations in glucose levels, which are known to precipitate migraines in susceptible individuals [14]. Low levels of physical activity, observed more frequently in the high migraine group, may reflect a cycle of migraine-related disability and reduced exercise tolerance [15]. However, regular moderate physical activity has been shown to reduce migraine frequency through endorphin release, improved sleep and reduced anxiety. Finally, increased screen time was correlated with higher migraine frequency, potentially due to prolonged exposure to blue light, poor posture and visual strain [16]. The clustering of these negative lifestyle behaviors in the high-frequency migraine group suggests a cumulative effect that could worsen migraine chronicity [17]. These findings have clinical implications: they support the integration of lifestyle assessments and behavioral interventions in the routine management of chronic migraine [18]. Chronic stress and poor mental health significantly contributed to migraine exacerbation, with stress management techniques proving beneficial. Environmental factors, including light, sound, weather changes, and allergens, were also identified as significant migraine triggers [19]. While pharmacologic treatments remain the mainstay, personalized counseling to improve sleep hygiene, stress reduction techniques, dietary regulation and physical activity can offer significant non-pharmacologic benefit [20]. Future longitudinal studies and randomized controlled trials are needed to establish causal relationships and to evaluate the effectiveness of lifestyle modification programs in reducing migraine burden.

## Conclusion:

This study establishes strong associations between modifiable lifestyle factors and chronic migraine frequency. Poor sleep quality, elevated stress, excessive caffeine intake, irregular meals, physical inactivity and prolonged screen time were significantly linked to higher monthly migraine days. These findings underscore the need for integrative, lifestyle-focused strategies in migraine management. Clinicians should routinely evaluate behavioral patterns and counsel patients on targeted modifications to reduce migraine burden and improve overall quality of life.
